# Supplementary material for: Extremely high upper critical field in BiCh2-based (Ch: S and Se) layered superconductor LaO0.5F0.5BiS2−xSex (x = 0.22 and 0.69)
Source: Sci Rep. 2022 Jan 7;12:288. doi: 10.1038/s41598-021-04393-3 (PMC8741910; doi:10.1038/s41598-021-04393-3)
Supplement: Supplementary file 1 — Supplementary Information. [file 41598_2021_4393_MOESM1_ESM.pdf]

Extremely high upper critical field in $BiCh_2$-based (Ch: S and Se) layered superconductor $LaO_{0.5}F_{0.5}BiS_{2-x}Se_x$ (x = 0.22 and 0.69)

Kazuhisa Hoshi[1], Ryosuke Kurihara[2], Yosuke Goto[1], Masashi Tokunaga[2], and Yoshikazu Mizuguchi[1]

[1]Department of Physics, Tokyo Metropolitan University, 1-1 Minami-osawa, Hachioji, Tokyo 192-0397, Japan

[2]The Institute for Solid-State Physics, University of Tokyo, 5-1-5 Kashiwanoha, Kashiwa, Chiba 277-8581, Japan

S.1 Angular dependence of B_{c2} at different temperatures from the main text

We investigated the angular dependence of the upper critical fields for x = 0.22 at 2.3 K and x = 0.69 at 3.3 K, respectively. The anisotropic 3D GL model is relatively fitted for the angular-dependent B_{c2}, which is consistent with Fig. 5 in the main text.

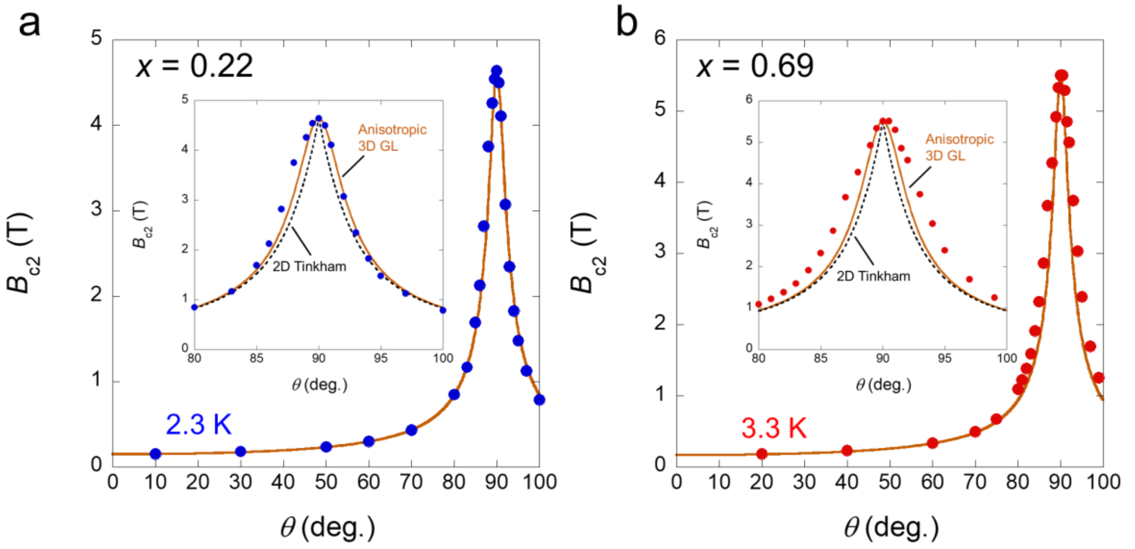

Supplementary Fig. 1 Three-dimensional (3D) nature of the upper critical field. **a, b** Angular θ dependence of the upper critical field at 2.3 K for x = 0.22 (**a**) and at 3.3 K for x = 0.69 (**b**). θ represents the angle between the c-axis and the direction of the applied magnetic field. The insets show magnified views of the region around θ = 90°. The solid curves denote the anisotropic 3D Ginzburg-Landau (GL) model. The dashed curves show the two-dimensional (2D) Tinkham's formula.

S.2 Estimation of B_{c2}

In this work, we estimated the upper critical fields as the midpoint of the resistive transition of the $\rho_{ab}(B)$ curves. Many studies have approved of this criterion as the upper critical field [1-6]. We show the criteria to estimate the upper critical fields in Supplementary Fig. 2. Moreover, we estimated upper critical fields from the temperature where resistivity begins to increase from zero resistivity. We show examples of the criteria as the beginning of the resistive increase from zero resistivity of the $\rho_{ab}(B)$ curves in Supplementary Fig. 3. The upper critical fields estimated from the Supplementary Fig. 3 are displayed in Supplementary Fig. 4. The observed upper critical fields for both $x = 0.22$ and $x = 0.69$ by the pulsed fields clearly exceed Pauli limits and deviate from WHH curves although the upper critical fields for $x = 0.22$ by static field (red closed circles in Supplementary Fig. 4a) are almost consistent with the WHH curve. The upper critical fields by static fields for $x = 0.69$ (red closed circles in Supplementary Fig. 4b) show upward behaviour, which is similar to Fig.4b in the main text.

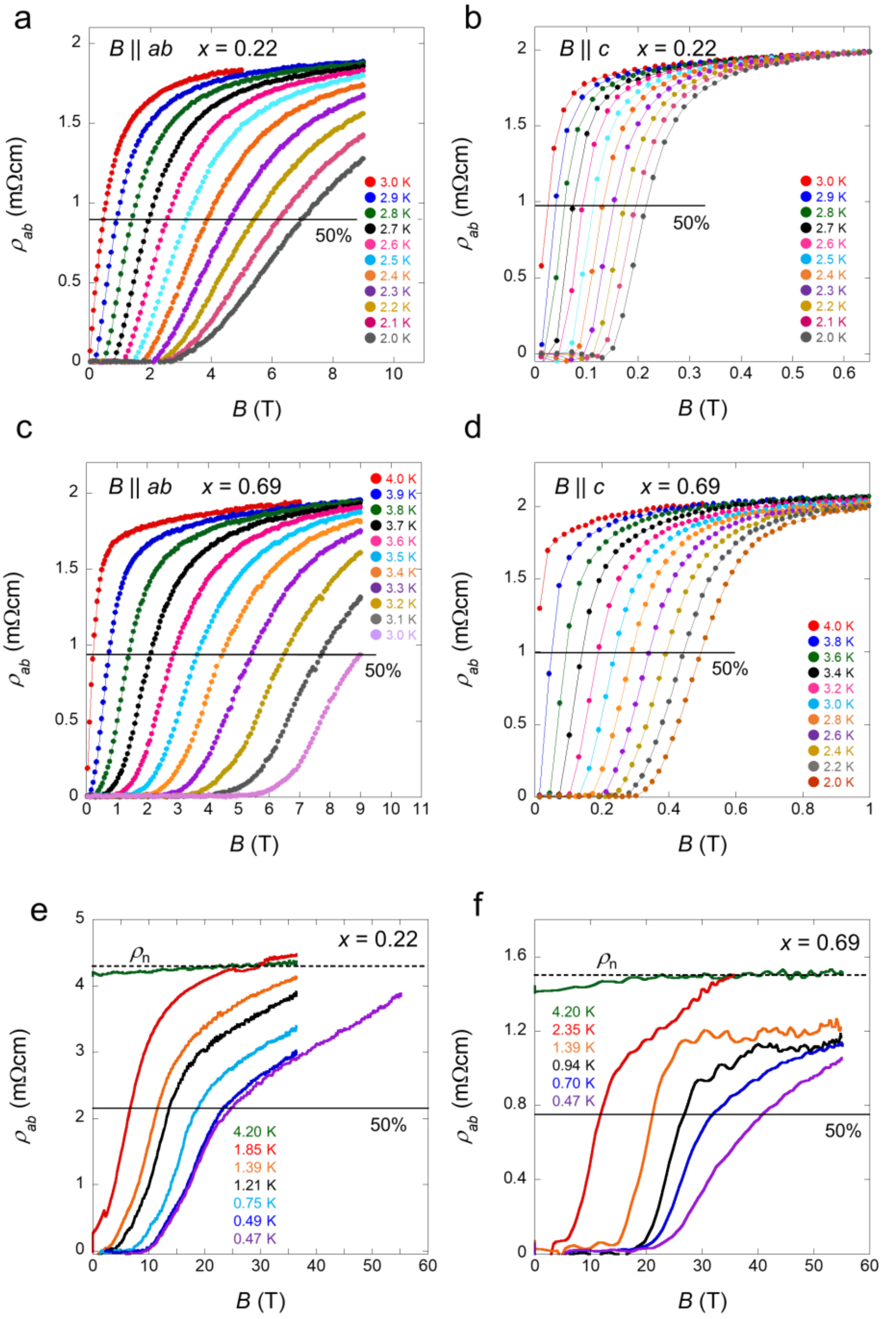

Supplementary Fig. 2 Estimation of B_{c2} form the midpoint of the resistive transition of the $\rho_{ab}(B)$ curves. **a**–**f** Field dependence of the resistivity by static field for $x = 0.22$ in the fields parallel to the ab-plane (**a**) and c-axis (**b**), for $x = 0.69$ in the fields parallel to the ab-plane (**c**) and c-axis (**d**), and by pulsed field for $x = 0.22$ (**e**) and $x = 0.69$ (**f**). The black solid lines of 50% indicate the midpoint of the resistive transition of the $\rho(B)$ curves to estimate the upper critical fields for Fig. 4 in the main text.

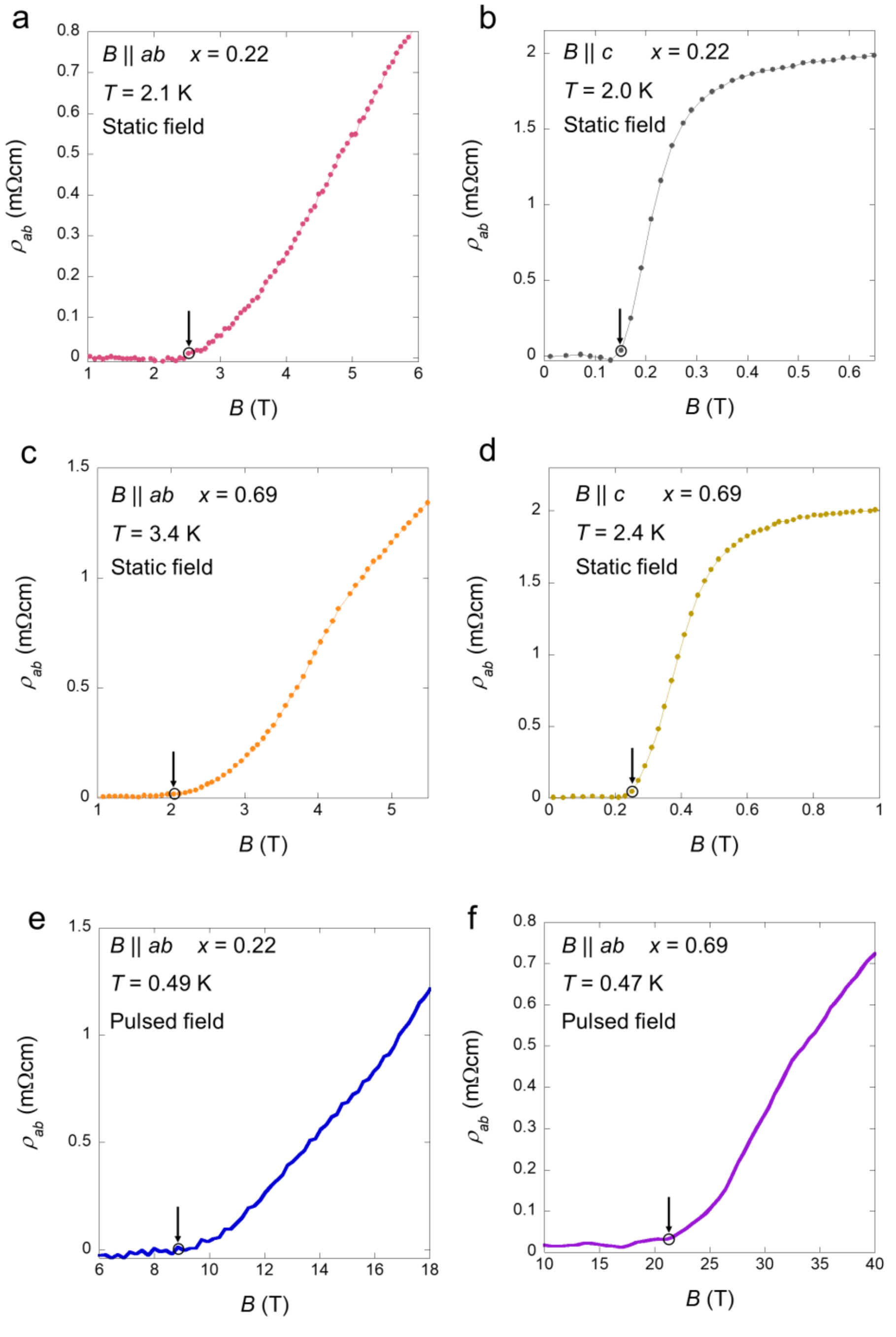

Supplementary Fig. 3 Examples of the criteria for the beginning of the resistive increase from zero resistivity of the $\rho_{ab}(B)$ curves. **a–f** Field dependence of the resistivity by static field for x = 0.22 in the fields parallel to the *ab*-plane (**a**) and *c*-axis (**b**), for x = 0.69 in the fields parallel to the *ab*-plane (**c**) and *c*-axis (**d**), and by pulsed field for x = 0.22 (**e**) and x = 0.69 (**f**). The black arrows and open circles exhibit the beginning of the resistive increase from zero resistivity of the $\rho_{ab}(B)$ curves to estimate the upper critical fields for Supplementary Fig. 4.

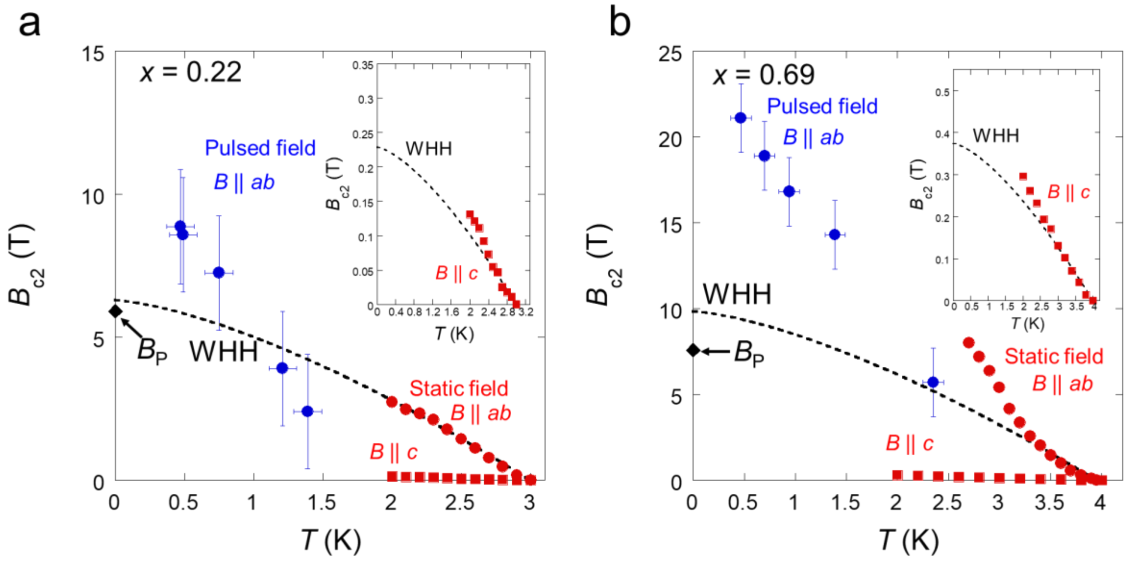

Supplementary Fig. 4 B_{c2} determined from the beginning of the resistive increase from the zero resistivity of the $\rho_{ab}(B)$ curves. **a, b** Temperature dependence of the upper critical fields estimated from the beginnings of the resistive increase from zero resistivity of the $\rho_{ab}(B)$ curves for $x = 0.22$ (**a**) and $x = 0.69$ (**b**).

S.3 Comparison with B_{c2} estimated from $\rho_{ab}(T)$ and $\rho_{ab}(B)$

In order to compare with the upper critical fields determined from $\rho_{ab}(T)$ and $\rho_{ab}(B)$ curves, we show various upper critical fields in Supplementary Fig. 5. The upper critical fields estimated from $\rho_{ab}(T)$ curves are almost consistent with $\rho_{ab}(B)$ curves.

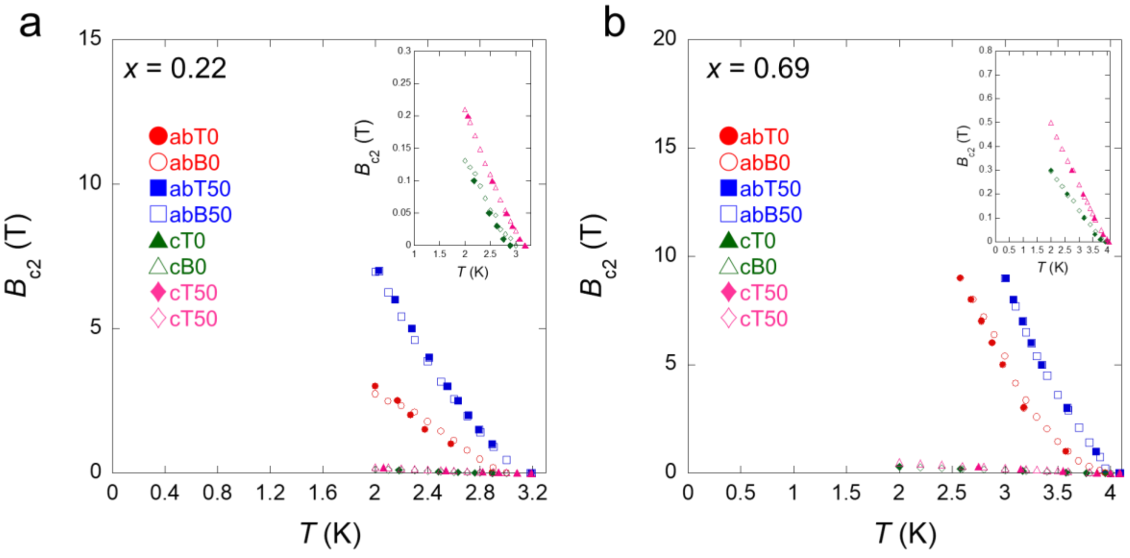

Supplementary Fig. 5 Comparison of B_{c2} estimated from $\rho_{ab}(T)$ and $\rho_{ab}(B)$ curves. **a, b** Temperature dependence of the upper critical fields from the $\rho_{ab}(T)$ and $\rho_{ab}(B)$ curves for $x = 0.22$ (**a**) and $x = 0.69$ (**b**). The abT0 (red closed circles) shows the in-plane upper critical fields defined as the beginning of the resistive increase from zero resistivity of the $\rho_{ab}(T)$ data, the abB0 (red open circles) of the $\rho_{ab}(B)$ data, abT50 (blue closed squares) as the midpoint of the resistive transition of the $\rho_{ab}(T)$ and abB50 (blue open squares) of the $\rho_{ab}(B)$, respectively. The cT0, cB0, cT50, and cT50 show the out-of-plane upper critical fields from same way as the in-plane upper critical fields.

Supplementary references

[1] Saito, Y. et al. Superconductivity protected by spin-valley locking in ion-gated MoS_2. *Nat. Phys.* **12**, 144–149 (2016).

[2] Lu, J. M. et al. Evidence for two-dimensional Ising superconductivity in gated MoS2. Science 350, 1353–1357 (2015).

[3] Mizukami, Y. et al. Extremely strong-coupling superconductivity in artificial two-dimensional Kondo lattices. *Nat. Phys.* **7**, 849–853 (2011).

[4] Goh, S. K. et al. Anomalous upper critical field in $CeCoIn_5/YbCoIn_5$ superlattices with a Rashba-type heavy Fermion interface. *Phys. Rev. Lett.* **109**, 157006 (2012).

[5] Chan, Y.-C. et al. Anisotropic two-gap superconductivity and the absence of a Pauli paramagnetic limit in single-crystalline $LaO_{0.5}F_{0.5}BiS_2$. *Phys. Rev. B* **97**, 104509 (2018).

[6] Pan, Y. et al. Rotational symmetry breaking in the topological superconductor SrxBi2Se3 probed by upper-critical field experiments. Sci. Rep. 6, 28632 (2016).
